# The Animal Model Determines the Results of *Aeromonas* Virulence Factors

**DOI:** 10.3389/fmicb.2016.01574

**Published:** 2016-10-04

**Authors:** Alejandro Romero, Paolo R. Saraceni, Susana Merino, Antonio Figueras, Juan M. Tomás, Beatriz Novoa

**Affiliations:** ^1^Department of Immunology and Genomics, Marine Research Institute-Consejo Superior de Investigaciones Científicas, VigoSpain; ^2^Department of Microbiology, Faculty of Biology, University of Barcelona, BarcelonaSpain

**Keywords:** animal model, zebrafish larvae, rainbow trout, mice, *Aeromonas*, virulence factors, *in vivo* infection, mutant *Aeromonas*

## Abstract

The selection of an experimental animal model is of great importance in the study of bacterial virulence factors. Here, a bath infection of zebrafish larvae is proposed as an alternative model to study the virulence factors of *Aeromonas hydrophila*. Intraperitoneal infections in mice and trout were compared with bath infections in zebrafish larvae using specific mutants. The great advantage of this model is that bath immersion mimics the natural route of infection, and injury to the tail also provides a natural portal of entry for the bacteria. The implication of T3SS in the virulence of *A. hydrophila* was analyzed using the AH-1::*aopB* mutant. This mutant was less virulent than the wild-type strain when inoculated into zebrafish larvae, as described in other vertebrates. However, the zebrafish model exhibited slight differences in mortality kinetics only observed using invertebrate models. Infections using the mutant AH-1ΔvapA lacking the gene coding for the surface S-layer suggested that this protein was not totally necessary to the bacteria once it was inside the host, but it contributed to the inflammatory response. Only when healthy zebrafish larvae were infected did the mutant produce less mortality than the wild-type. Variations between models were evidenced using the AH-1ΔrmlB, which lacks the O-antigen lipopolysaccharide (LPS), and the AH-1ΔwahD, which lacks the O-antigen LPS and part of the LPS outer-core. Both mutants showed decreased mortality in all of the animal models, but the differences between them were only observed in injured zebrafish larvae, suggesting that residues from the LPS outer core must be important for virulence. The greatest differences were observed using the AH-1ΔFlaB-J (lacking polar flagella and unable to swim) and the AH-1::motX (non-motile but producing flagella). They were as pathogenic as the wild-type strain when injected into mice and trout, but no mortalities were registered in zebrafish larvae. This study demonstrates that zebrafish larvae can be used as a host model to assess the virulence factors of *A. hydrophila*. This model revealed more differences in pathogenicity than the *in vitro* models and enabled the detection of slight variations in pathogenesis not observed using intraperitoneal injections of mice or fish.

## Introduction

*Aeromonas hydrophila* is a Gram-negative, motile, rod-shaped bacterium widely distributed in aquatic environments (Janda and Abbott, 2010). It is the most common opportunistic species of *Aeromonas* that causes infections in humans. Transmission to humans occurs mainly by water because its natural residence in aquatic environments favors its appearance in drinking water and food. Infections of *A. hydrophila* produce gastrointestinal disorders (Chopra and Houston, 1999). Additionally, infections caused by the exposure of opened wounds to contaminated water resulted in cellulitis of the subcutaneous tissues (Janda and Abbott, 2010). Immunocompromised people with cancer, hepatic diseases, diabetes or trauma have a higher risk of developing sepsis and fatal *A. hydrophila* infections (Parker and Shaw, 2011). Other animals, such as other mammals, birds, reptiles, amphibians, and fish, can also be infected (Fulton, 1965; Esterabadi et al., 1973; Cipriano et al., 1984; Gray, 1984; Glunder and Siegmann, 1989; Rodríguez et al., 2008; Hill et al., 2010; Schadich and Cole, 2010). In many freshwater fish species (e.g., carp, catfish, eels, and golden fish), it produces motile haemorrhagic septicaemia (MAS), which causes high mortality rates in aquaculture farms and, in turn, large economic losses (Cipriano et al., 1984). *A. hydrophila* is naturally present in the gut microbiota of zebrafish (Cantas et al., 2012), and it has been able to generate acute infection in adults (Rodríguez et al., 2008) and embryos (Saraceni et al., 2016) under controlled experimental conditions. *A. hydrophila* is also infective in invertebrates, such as crustaceans (Jiravanichpaisal et al., 2009), mealworms (Noonin et al., 2010) and unicellular organisms such as *Dictyostelium* amoebae (Froquet et al., 2007).

The main pathogenic factors associated with *Aeromona*s are surface polysaccharides (capsule, lipopolysaccharide, and glucan), S-layers, iron-binding systems, exotoxins, extracellular enzymes, secretion systems, fimbriae and other non-filamentous adhesins, motility and flagella (see review by Tomás, 2012).

In the last few years, numerous *in vitro* and *in vivo* experiments have been conducted to analyze the specific role of each virulence factor in the pathogenesis of *A. hydrophila*, using several mutant strains and purified/recombinant toxins. *In vitro* studies have used cell lines to evaluate the immune response triggered by *A. hydrophila* by measuring phagocytosis and respiratory bursts (Fadl et al., 2007; Reyes-Becerril et al., 2011). In addition, several bacterial phenotypes related to adhesion and invasion of cells, serum resistance and cytotoxic activity have also been evaluated (Merino et al., 1997; Vilches et al., 2007). The great limitation of these *in vitro* experiments is the lack of the tissue context that definitely influences the evolution of the infection.

*In vivo* study of *Aeromonas* virulence factors has classically been conducted in mice because their immune defense system is similar to that of humans. In this model, the bacterium is usually parenterally administered by intramuscular or intraperitoneal injection (Sha et al., 2005; Yu et al., 2005; Chen et al., 2014) or orally by deposition of the bacteria in water (Wong et al., 1996). Injection routes of infection afford the bacteria full access to the animal without the involvement of modified virulence factors. The use of new animal models is being explored (Froquet et al., 2007) because vertebrate animal models of infection are costly and have raised ethical issues. Moreover, the results from mice cannot be applied to bacteria that infect cold-blooded vertebrates living at low temperatures.

Invertebrate host models have been developed and are being used to study the virulence of human bacterial pathogens (Kurz and Ewbank, 2007; O’Callaghan and Vergunst, 2010). The species used range from terrestrial invertebrates, such as nematodes and insects, to freshwater and marine life, including planarians, crustaceans, molluscs, and many others. In particular, the nematode *Caenorhabditis elegans* and insects such as the greater wax moth *Galleria mellonella* and the fruit fly *Drosophila* have been used to identify the virulence factors of *Pseudomonas aeruginosa* (Lutter et al., 2008; Garvis et al., 2009; Ramarao et al., 2012) and to analyze clinical isolates of human bacteria (see review O’Callaghan and Vergunst, 2010). Invertebrates such as *Pacifastacus leniusculus* (crayfish), *Tenebrio molitor* larvae (mealworms), and even the unicellular organisms *Acanthamoeba castellanii* and *Dictyostelium discoideum* amoebae (Froquet et al., 2007; Noonin et al., 2010) have also proved useful for studying bacteria virulence factors (Goy et al., 2007; Kurz and Ewbank, 2007).

*Aeromonas hydrophila* and *A. veronii* are the predominant bacterial flora in the gut of the leech, where they play essential roles for the animal in the digestion of blood (Bickel et al., 1994). Human soft tissue infections with this bacterium have been increasing due to the use of medicinal leeches (*Hirudo medicinalis* and *H. verbana*) for the treatment of venous congestion in flaps and replanted parts (Maetz et al., 2007). The symbiotic association between *Aeromonas* and medical leeches has allowed for the use of this invertebrate as a model for digestive tract associations (Graf, 1999; Braschler et al., 2003; Graf et al., 2006). Bacterial virulence factors, such as secretion systems type 2 (T2SS) and type 3 (T3SS) of *A. veronii*, have been studied in the leech model, suggesting that the bacteria use known virulence factors in a manner that allow them to colonize and persist in the leech crop without causing any apparent negative effects (reviewed in Nelson and Graf, 2012). The feasibility of these invertebrate models is based on the low species specificity of the pathogens due to the universality of virulence factors implicated in the infectious process (Froquet et al., 2007).

In the last few years, non-mammalian host models such as fish, particularly zebrafish (*Danio rerio*), have been used as infection models to study bacterial infections such as those produced by *Streptococcus, Salmonella*, or *Mycobacterium* (Neely et al., 2002; van der Sar et al., 2003; Swaim et al., 2006), as well as viral infections produced by rhabdoviruses or Herpes viruses (Burgos et al., 2008; Ludwig et al., 2011; Varela et al., 2014). Adult zebrafish and other fish species, such as rainbow trout and blue gourami, have also been used to study virulence factors of *A. hydrophila* (Yu et al., 2004, 2005; Sha et al., 2005; Canals et al., 2006a, 2007a; Vilches et al., 2007). These fish models offer important advantages. The ease of use and low costs for obtaining large numbers of animals allow for large-scale screening that would be prohibitive in mammals.

In the present work, zebrafish larvae are presented as an alternative animal model to study the virulence factors of *A. hydrophila* using specific mutants of this bacterium. Experimental infections in zebrafish were compared with similar experiments using classical models, such as mice and adult rainbow trout. The great advantage of the zebrafish larvae over other models is that experimental infection by bath immersion mimics the natural route of bacterial infection. Moreover, an injury in the tail also provides a natural portal of entry for the bacteria by mimicking the wounds that are frequently used as a portal to spread infection. The importance of the selection of the right animal model to study virulence factors is discussed.

## Materials and Methods

### Bacterial Strains, Plasmids, and Growth Conditions

The bacterial strains and plasmids used in this study are listed in **Table 1**. *Escherichia coli* strains were grown on Luria-Bertani (LB) Miller broth and LB Miller agar at 37°C, while *A. hydrophila* strains were grown either in tryptic soy broth (TSB) or agar (TSA) at 30°C. Spectinomycin (100 μg⋅mL^-1^), rifampicin (100 μg⋅mL^-1^), chloramphenicol (12.5 μg⋅mL^-1^), and kanamycin (25 μg⋅mL^-1^) were added to the different media when required.

**Table 1:** Bacterial strains and plasmids used in this study.

Strain or plasmid	Relevant characteristics	Reference
***E. coli* strains**		
DH5α	F^-^ *end A hsdR17* (rK^-^ mK^+^) *supE44 thi-1 recA1 gyr-A96*_*80lacZ*M15	Hanahan, 1983
BL21(λD3)	F^-^ *ompT hsdS_B_* (r_B_^-^ m_B_^-^) *gal dcm*(λD3)	Novagen
MC1061λpir	*thi thr1 leu6 proA2 his4 argE2 lacY1 galK2 ara14 xyl5 supE44*λ *pir*	Canals et al., 2006b
***A. hydrophila* strains**		
AH-1	O11, wild-type	Yu et al., 2004
AH-1 Rif^R^	Wild-type, rifampicin resistance	Yu et al., 2004
AH-1::*aopB*	AH-1 *aopB* defined insertion mutant, Cm^R^, T3SS^-^	Yu et al., 2004
AH-1ΔRmlB	AH-1 *rmlB* mutant in frame unable to produce O11-antigen LPS	Merino et al., 2015
AH-1ΔWahD	AH-1 *wahD* mutant in frame unable to produce O11-antigen LPS and part of the LPS outer core	This work
AH-1ΔvapA	AH-1 mutant in frame unable to produce S-layer	Merino et al., 2015
AH-1 ΔFlaB-J	AH-1 *flaB* to *J* deleted mutant in frame unable to produce polar flagellum	This work
AH-1::MotX	AH-1 *motX* defined insertion mutant, Cm^R^, non-motile	This work
**Plasmids**		
pRK2073	Helper plasmid, Sp^R^	Jimenez et al., 2008
pDM4	Suicide plasmid, Sacarose, Cm^R^	Milton et al., 1996
pDM4-WahD	pDM4 with truncated AH-3 *wahD*, also pDM4Δ5.1	Jimenez et al., 2008
pDM4-FlaB-J	pDM4 with truncated AH-1 *flaB* to *J*	This work
pFS-MotX	pFS100 with an internal fragment of AH-3 *motX*, Km^R^.	Canals et al., 2006b

### Construction of Defined Mutants

DNA probes from polar flagella region 2 of *A. hydrophila* AH-3 (Canals et al., 2006b) (actually classified as *A. piscicola* by Beaz-Hidalgo et al., 2009) allowed for identification of a clone from a cosmid genomic library of *A. hydrophila* AH-1 (Merino et al., 2015). This clone allowed us to use the DNA sequence of complete region 2 (Canals et al., 2006b) of AH-1 and also this DNA sequence for mutant isolation. Mutant AH-1ΔwahD was obtained using plasmid pDM4-wahD generated in strain AH-3, as indicated previously (Jimenez et al., 2008). WahD is the glycosyltransferase that adds D-D-Hep to Glc in an α 1→6 linkage (Jimenez et al., 2008).

The chromosomal in-frame flaB-J and wahD deletion mutants *A. hydrophila* AH-1ΔFlaB-J and AH-1ΔWahD, respectively, were constructed by allelic exchange as described by Milton et al. (1996). Briefly, upstream (fragment AB) and downstream (fragment CD) fragments of flaB-J were independently amplified using two sets of asymmetric PCRs. Primer pairs A-FlaB (5′-CGG GATCCAACAGTCTG CCAATGGTTC-3′), B-FlaB (5′-CCC ATCCACTAAACTTAAACAGTTAGCCTGAGCCAAAATG-3′), C-FlaJ (5′-TGTTTAAGTTTAGTGGATGGGAGACAACAGCTA GGGGAGTT-3′) and D-FlaJ (5′-CGGGATCCAACGTTTCAC AAGCAAGA-3′) amplified DNA fragments of 581 (AB) and 637 (CD) bp for flaB-J in-frame deletion. DNA fragments AB and CD were annealed and amplified as a single fragment using primers A and D. The fusion product was purified, and BamHI was digested and ligated into BglII-digested and phosphatase-treated pDM4 vector (Milton et al., 1996); then, it was electroporated into *E. coli* MC1061 (λpir) and was plated on chloramphenicol plates at 30°C to obtain pDM4-FlaB-J. Plasmid pDM4-WahD (formerly pDM4Δ5.1) was previously obtained (Jimenez et al., 2008). Plasmids pDM4 with mutated genes was transferred into *A. hydrophila* AH-1 Rif^R^ by triparental mating, using *E. coli* MC1061 (λpir) containing the insertion constructs and the mobilizing strain HB101/pRK2073. Transconjugants were selected on plates containing chloramphenicol and rifampicin. PCR analysis confirmed that the vector had integrated correctly into the chromosomal DNA. After sucrose treatment, transconjugants that were rifampicin resistant (Rif ^R^) and chloramphenicol sensitive (Cm^S^) were chosen and confirmed by PCR, obtaining *A. hydrophila* AH-1ΔFlaB-J and AH-1ΔWapD mutants.

The insertional defined mutant AH-1::motX was constructed using the plasmid construction of strain AH-3 (pSF-MotX) by a single recombination event, leading to the generation of two incomplete copies of motX in the chromosome of the mutant, as previously described (Canals et al., 2006b). Plasmid pSF-MotX was isolated, transformed into *E. coli* MC1061(λpir), and transferred by conjugation from *E. coli* MC1061 to the *A. hydrophila* strain AH-1 Rif^R^, as previously described (Canals et al., 2006b). Km^r^ Rif^r^ transconjugants arising from pSF-MotX should contain the mobilized plasmid integrated onto the chromosome by homologous recombination between the motX and the plasmid, leading to two incomplete copies of the motX (defined insertion mutant). Chromosomal DNA from transconjugants obtained was analyzed by Southern blot hybridization with an appropriate motX DNA probe to obtain the defined insertion AH-1::motX mutant, as previously described (Canals et al., 2006b).

### Motility Assays

Freshly grown bacterial colonies (mutants AH-1ΔflaB-J and AH-1::motX) were transferred with a sterile toothpick into the center of swim agar (1% tryptone, 0.5% NaCl, 0.25% agar). The plates were incubated face up for 16–24 h at 30°C, and motility was assessed by examining the migration of bacteria through the agar from the center toward the periphery of the plate. Motility was also assessed by light microscopy observations in liquid media.

### Transmission Electron Microscopy (TEM)

Bacterial suspensions (mutants AH-1ΔflaB-J and AH-1::motX) were placed on Formvar-coated grids and were negatively stained with a 2% solution of uranyl acetate at a pH of 4.1. The preparations were observed on a Hitachi 600 transmission electron microscope (Hitachi High-Technologies Corp. Tokyo, Japan).

### LPS Isolation and SDS-PAGE

For screening purposes, lipopolysaccharide (LPS) was obtained after proteinase K digestion of whole cells (Hitchcock and Brown, 1983). LPS samples were separated by SDS-PAGE and were visualized by silver staining, as previously described (Tsai and Frasch, 1982; Hitchcock and Brown, 1983). For large-scale isolation, LPS was extracted from dried bacterial cells with aqueous 45% phenol at 68°C by the phenol/water method (Westphal and Jann, 1965). For sugar analysis, a polysaccharide sample (0.5 mg) was hydrolysed with 2 M CF_3_CO_3_H (100°C, 4 h), the monosaccharides were conventionally converted into the alditol acetates and analyzed by gas liquid chromatography (GLC) on a Varian 3700 chromatograph (Santa Clara, CA, USA), equipped with a fused-silica gel SPB-5 column using a temperature gradient from 150°C (3 min) to 320°C at 5°C min^-1^.

### S-layer Purification and Zebrafish Stimulation

The S-layer sheet material was obtained from the AH-1ΔrmlB mutant (O-antigen negative) as briefly described: cells were grown overnight in 1000 mL of Luria Broth (LB) with agitation (200 rpm), harvested by centrifugation (12,000 × *g*, 20 min), and washed twice in 20 mM Tris-HCl (pH 8.0). They were suspended in 100 mL of 0.2 M glycine-HCl (pH 2.8) and were stirred at 4°C for 30 min. The cells were removed by a single centrifugation at 12,000 × *g* for 20 min. The S-layer sheet material was collected by centrifugation at 40,000 × *g* for 60 min, suspended in 500 mL of 20 mM Tris-HCl (pH 8.0), and frozen at -20°C.

Zebrafish larvae (4 days post fertilization) were microinjected in the Duct of Cuvier with 100 ng of the purified S-layer. The animals were sampled at 3 and 24 h after stimulation to analyze the gene expression, using the protocol described below in section “Quantitative PCR.”

### DNA Manipulations

General DNA manipulations were performed essentially as previously described (Sambrook et al., 1989). DNA restriction endonucleases, T4 DNA ligase, *E. coli* DNA polymerase (Klenow fragment), and alkaline phosphatase were used as recommended by Sigma–Aldrich (St. Louis, MO, USA). Double-stranded DNA sequencing was performed using the dideoxy-chain termination method (Sanger et al., 1977) from PCR-amplified DNA fragments with the ABI Prism dye terminator cycle sequencing kit (PerkinElmer, Barcelona, Spain). Oligonucleotides used for genomic DNA amplifications and DNA sequencing were purchased from Sigma–Aldrich (St. Louis, MO, USA). Deduced amino acid sequences were compared with those of DNA translated in all six frames from non-redundant GenBank and EMBL databases, using the BLAST (Altschul et al., 1997) network service at the National Center for Biotechnology Information and the European Biotechnology Information.

### Virulence for Fish and Mice

The virulence of the strains grown at 30°C was measured by monitoring their 50% lethal doses (LD_50_) by the method of Reed and Muench (1938), using different animal models.

Two fish species, rainbow trout (*Oncorhynchus mykiss*) and zebrafish (*Danio rerio*), were used to evaluate the virulence of the different mutant strains.

Rainbow trout (15 g mean) were maintained in 20-liter static tanks at 17–18°C. The fish were injected intraperitoneally with 0.05 mL of the test samples (approximately 10^9^ viable cells), and the mortality was recorded for up to 2 weeks. Three independent experimental infections were conducted. All of the deaths occurred within 2 to 8 days.

Healthy and injured zebrafish larvae (4 dpf) were infected following the protocol described by Saraceni et al. (2016). Injured larvae were obtained by complete transection of the tail fin, using a sapphire blade under an SMZ800 stereomicroscope (Nikon). Groups of ten healthy and injured larvae were distributed into 6-well plates (Falcon) containing 6 mL of sterile E3 eggs in water. For infection, the bacteria were resuspended in phosphate-buffered saline (PBS) and were added to each well to reach a final concentration of 10^7^–10^8^ CFU·mL^-1^ and incubated at 28°C. The inoculated bacteria were kept in the water during all of the experiments. Control groups were treated with the same volume of PBS. Cumulative mortalities were registered until 120 hpi. All of the experimental infections were performed five times using four biological replicates of 10 larvae each.

Albino Swiss female mice (5–7 weeks old) were injected intraperitoneally with 0.25 ml of the test samples (approximately 5 × 10^9^ viable cells). Mortality was recorded for up to 1 week. Three independent experimental infections were conducted. All of the deaths occurred within 2–5 days.

The protocol estimates several possibilities to sacrifice the animals: when they lose more than 20% of their weights, when a characteristic pain position is observed (number 3 in our rating), signs of coma, or any self-mutilation. Mortality was considered to be caused by the bacterium only if inoculated bacteria were recovered from the studied dead animals. The animals were monitored twice per day and were sacrificed by asphyxiation in a CO_2_ atmosphere at the end of the experiment or using humane endpoints. No animals involved in the LD_50_ testing died without human intervention.

### Quantitative PCR

Total larvae RNA was automatically extracted using the Maxwell^®^ 16 LEV simply RNA Tissue kit (Promega), according to the manufacturer’s instructions. First-strand cDNAs were synthesized using SuperScript II (Life Technologies). The expression of the IL-1β gene was measured by qPCR following the protocol previously described by Pereiro et al. (2015). The elongation factor gene (zEF1) was used as a housekeeping gene to normalize the expression values because it has stable expression that does not change with infection. Fold-change units were calculated by dividing the normalized expression values of the infected larvae by the normalized expression values of the controls. Two independent experiments of 3 biological replicates each were performed.

### Bacterial Burden in Infected Zebrafish

To evaluate the ability of bacterial mutants to induce a stable infection, the evolution of the bacterial burden was analyzed in infected injured zebrafish at 1 and 6 hpi. At each time point, four groups of 10 larvae were anesthetized with a lethal dose of MS-222 (Sigma–Aldrich), transferred to a tube containing 200 μL of 1% Triton-X100 (BIO-RAD) and mechanically homogenized. Serial dilutions of the homogenates were prepared in PBS and were plated in selective TSA plates. Colony-forming units (CFU) were counted after overnight incubation at 28°C in two independent experiments.

### *In vitro* Effects of Mutant Strains

The effect of bacterial infection was assayed *in vitro* using primary cultures of the ZF-4 zebrafish cell line. This experiment was conducted three times using three cell cultures each. To evaluate cytotoxicity, bacterial mutants were also added to ZF4 monolayers.

### Statistical Analysis

Kaplan–Meier survival curves for the zebrafish infection experiments were analyzed with the log rank (Mantel-Cox) test. Multiple-comparison ANOVA and Tukey’s HSD test were conducted to analyze the evolution of Il-1β gene expression and the bacterial burden. The results are presented as the means ± standard errors of means (SEMs).

### Ethics Statement

The animal care and experimental infections were performed according to EU guidelines^1^. All of the experiments were performed by specialized technicians from the CSIC and the Faculty of Biology at the University of Barcelona, under the supervision of a veterinarian. All of the protocols were revised and approved by the Committees on Bioethics from the CSIC (151/2014) and by the Ethics Committee of the University of Barcelona (Permit Numbers: 4211 for fish and 4212 for mice).

## Results

### Importance of the T3SS in the Pathogenesis of *A. hydrophila*

**FIGURE 1:**
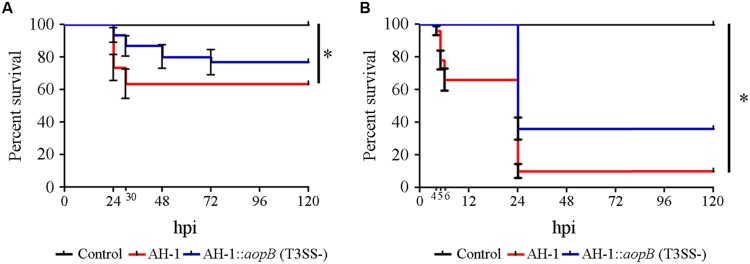
**Implications of the T3SS in the pathogenesis of *Aeromonas hydrophila*. (A)** Kaplan–Meier survival curve of healthy larvae after infection with bacterial suspensions. *Significant differences at *P* < 0.005. **(B)** Kaplan–Meier survival curve of injured larvae after infection with bacterial suspensions. *Significant differences at *P* < 0.0001. In all cases, the graphs show representative results of five independent experimental infections conducted using four biological replicates of ten larvae each. Healthy and injured larvae were infected with a bacterial suspension (AH-1 wild-type or AH-1::*aopB*) containing 2 × 10^7^ CFUs·mL^-1^ and 5 × 10^7^ CFUs·mL^-1^, respectively.

The mutant AH-1::*aopB* lacking a functional T3SS was generated to analyze the implications of this virulence factor in the pathogenesis of *A. hydrophila*. Sixty-three per cent of the healthy zebrafish larvae infected with the AH-1 wild-type strain survived at the end of the experiment. Mortality started at 24 hpi and increased until 30 hpi. The infection of sibling animals with the mutant AH-1::*aopB* lacking the T3SS induced significantly lower mortality levels than the wild-type; 80% of the animals survived to the end of the experiment (**Figure 1A**). When injured zebrafish larvae were infected, the AH-1 strain induced the death of almost 90% of the larvae at 120 hpi. The rate of survival registered in injured larvae infected with the mutant AH-1::*aopB* was significantly higher (36%), and the mortality was delayed until 24 h (**Figure 1B**). Similar results were obtained in the five experimental infections conducted.

### Evaluation of the O-antigen LPS in the Pathogenesis of *A. hydrophila*

**FIGURE 2:**
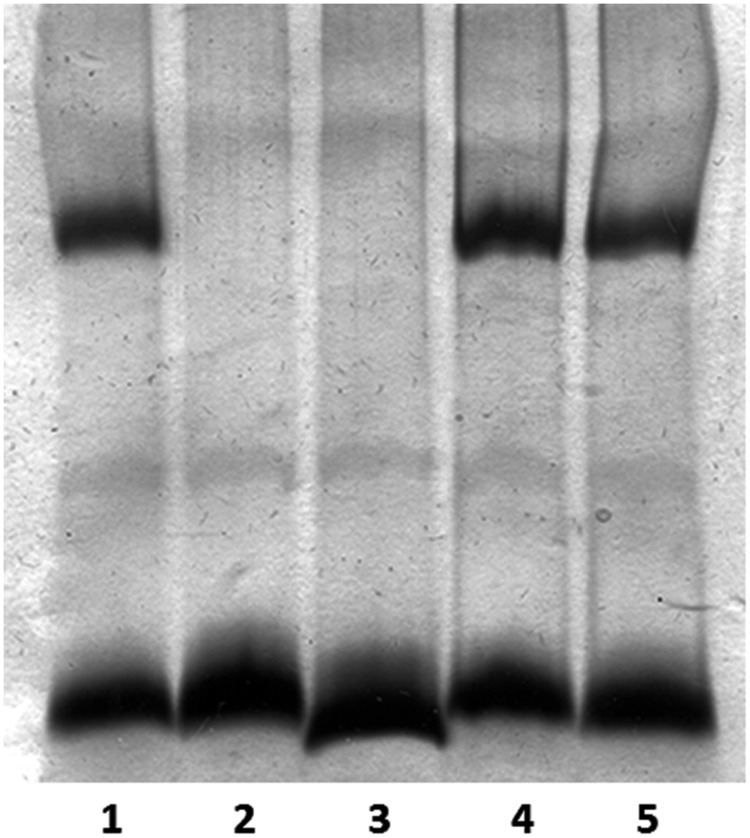
**Lipopolysaccharide (LPS) analyzed by SDS-Tricine gel and silver stained for *A. hydrophila* strains: AH-1 wild-type (lane 1), AH-1ΔrmlB mutant (lane 2), AH-1ΔwahD mutant (lane 3), AH-1ΔrmlB mutant complemented with pBAD-rmlB (lane 4), and AH-1ΔWahD mutant complemented with pBAD-wahA (lane 5)**.

The lipopolysaccharide mutants used in this study were AH-1ΔrmlB, which is devoid of the O-antigen LPS with a complete LPS-core, and AH-1ΔwahD, which lacks the O-antigen LPS and part of the LPS outer core. Analysis of purified LPS from mutant AH-1ΔwahD by GLC indicated that no D-D-Hep could be found, as well as their increased mobility from wild-type or AH-1ΔRmlB mutants in LPS gels, in agreement with the loss of part from the LPS outer core (**Figure 2**).

Experimental infections of mice and rainbow trout with the mutants (AH-1ΔrmlB and AH-1ΔwahD) resulted in a significantly increased LD_50_ compared to the lethal dose using the AH-1 wild-type strain. Infected mice died within 2–5 days post-infection, and the LD_50_ changed from 10^6.7^ using AH-1 wild-type to >10^8.0^ using mutant strains. In rainbow trout, deaths occurred within 2–8 days, and LD_50_ was also increased in mutant strains (**Table 2**).

**Table 2:** LD_50_ for rainbow trout and mice of *Aeromonas hydrophila* AH-1 and its mutants upon intraperitoneal injection of strains grown in TSB at 20°C to infect fish and at 37°C to infect mice.

Strain	Rainbow trout	Mice
AH-1 wild-type	10^4.5^	10^6.7^
AH-1ΔrmlB (O-antigen LPS^-^)	10^6.1^	>10^8.0^
AH-1ΔwahD (O-antigen and outer-core LPS^-^)	10^6.8^	>10^8.0^
AH-1ΔvapA (S-layer^-^)	10^4.6^	10^6.7^
AH-1ΔflaB-J (polar flagellum^-^)	10^4.4^	10^6.8^
AH-1::motX (motility^-^)	10^4.5^	10^6.6^

*The values are the averages from three independent experiments, and the maximum deviation was always <10^0.3^.*

**FIGURE 3:**
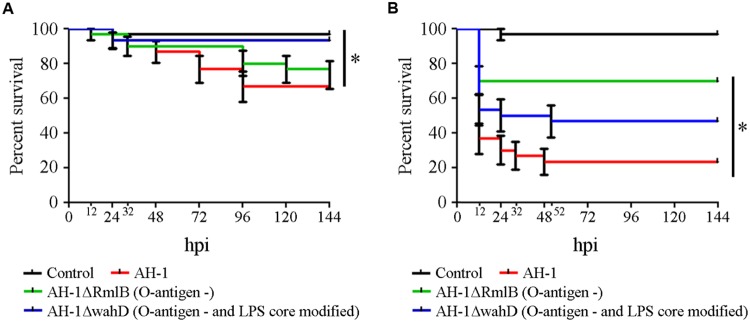
**Implications of the O-antigen in the pathogenesis of *A. hydrophila*. (A)** Kaplan–Meier survival curve of healthy larvae infected with *A. hydrophila* AH-1 wild-type and the mutants AH-1ΔRmlB (O-antigen negative) and AH-1ΔwahD (O-antigen negative and altered part of the LPS outer-core). *Significant differences at *P* = 0.0062. **(B)** Kaplan–Meier survival curve of injured larvae after infection with the same bacterial suspensions. *Significant differences at *P* < 0.0001.

In healthy zebrafish larvae, significantly higher survival rates were obtained in experimental infections with the mutants with changes in the O-antigen and in the LPS core compared to the survival rate obtained after infection with the wild-type bacteria (**Figure 3A**). The differences in survival rates between the AH-1 wild-type and the mutants were more evident when injured larvae were used for the infections (**Figure 3B**). In injured larvae, mortality started at 12 hpi regardless of the bacterial strain inoculated, and it resulted in significantly different survival rates according to the LPS modifications. Only 23% of the fish inoculated with the AH-1 wild-type survived. Modifications in the LPS induced significant increases in the survival rates at the end of the experiment. Forty-six percent of the fish inoculated with the mutant AH-1ΔwahD lacking the LPS-core and completely lacking the O-antigen survived, while 70% of fish infected with the mutant lacking O-antigen (AH-1ΔRmlB) were alive at 144 hpi (**Figure 3B**).

### Importance of the S-layer in the Pathogenesis of *A. hydrophila*

**FIGURE 4:**
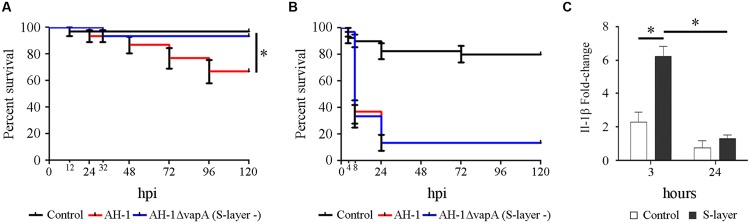
**Implications of the S-layer in the pathogenesis of *A. hydrophila*. (A)** Kaplan–Meier survival curve of healthy **(A)** and injured **(B)** larvae infected with *A. hydrophila* AH-1 wild-type and the mutant AH-1ΔvapA (S-layer negative). *Significant differences at *P* = 0.002. **(C)** Effects of microinjection of the purified AH-1 S-layer (100 ng) on the expression of Il-1β at 3 and 24 h post-injection. *Significant differences at *p* < 0.001.

Results obtained in experimental infections of mice and rainbow trout with the mutant AH-1ΔvapA (S-layer negative) showed that the mutant strain was as pathogenic as the wild-type. In both animal models, the LD_50_ obtained after ip injection was not different between the wild-type and mutant strains. The LD_50_ for mice and trout were 10^6.7^ and 10^4.6^, respectively (**Table 2**). In healthy zebrafish larvae, AH-1ΔvapA had low pathogenicity and produced less than 10% of the cumulative mortality. Ninety-three percent of infected fish survived at the end of the experiment. This percentage of survival was significantly higher than that registered in fish infected with the AH-1 wild-type strain, in which 66% of the fish survived the infection (**Figure 4A**). However, when injured larvae were infected, the mutant AH-1ΔvapA produced the same cumulative mortality levels as the AH-1 strain. The mortality started as soon as 8 hpi and reached the maximum value (up to 80%) at 24 hpi. Only 13% of the fish survived the bacterial infection (**Figure 4B**). The pro-inflammatory activity of the purified S-layer was assayed by qPCR to measure the Il-1β gene expression. The treatment of fish with the purified S-layer induced a significant increase in Il-1β gene expression at 3 hpi, reaching fold changes as much as six times greater than the control group. The expression level registered at 24 h was significantly lower than that registered at 3 h, but no significant differences were observed between the control and stimulated fish at this time (**Figure 4C**).

### Implications of the Motility and Polar Flagella in the Pathogenesis of *A. hydrophila*

**FIGURE 5:**
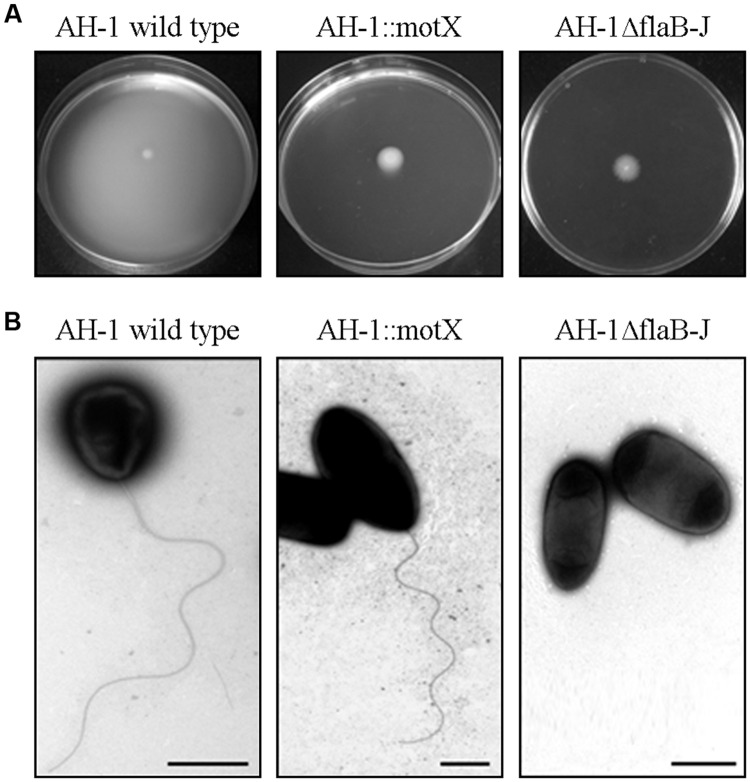
**(A)** Motility swimming in semisolid agar plates of the strain AH-1 wild-type, AH-1::motX mutant (no motility), and AH-1ΔflaB-J mutant (polar flagella negative). **(B)** Transmission electron microscopy (TEM) of *A. hydrophila* strains. Bacteria were gently placed onto Formvar-coated copper grids and were negatively stained using a 2% solution of uranyl acetate. Bar = 1 μm.

To analyze the implications of the bacterial motility and polar flagella in the pathogenesis of *A. hydrophila*, two mutants were generated. The AH-1ΔflaB-J mutant lacked a polar flagellum and was not able to swim, and the AH-1::motX mutant was non-motile but was able to produce a polar flagellum (**Figure 5**).

**FIGURE 6:**
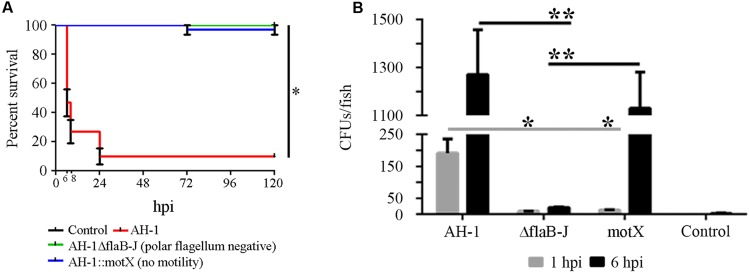
**Implication of the polar flagellum and the motility in the pathogenesis of *A. hydrophila*. (A)** Kaplan–Meier survival curve of injured larvae infected with *A. hydrophila* AH-1 wild-type and the mutants AH-1ΔflaB-J (polar flagellum negative) and AH-1::motX (no motility). *Significant differences at *P* < 0.0001. **(B)** Bacterial burden of infected larvae at 1 and 6 hpi. Mean ± SEM of two independent experiments are presented (*n* = 10 larvae per group). *p < 0.05, **p < 0.01.

When these mutants were injected into rainbow trout or mice, no differences in the LD_50_ of the mutants AH-1ΔflaB-J or AH-1::motX, compared to wild-type strain, could be observed (**Table 2**). Surprisingly, when zebrafish were infected with the mutants AH-1ΔflaB-J or AH-1::motX, no mortality was registered in either healthy (data not shown) or injured larvae (**Figure 6A**).

After infection, the bacterial load in fish significantly increased from 1 to 6 hpi in the fish infected with the AH-1 wild-type bacteria and those infected only with the mutant AH-1::motX (no motility). The bacterial concentration of mutant AH-1ΔflaB-J, which was also not motile because it lacked the polar flagellum, did not increase during the experiment and was maintained at a low level (**Figure 6B**).

### *In vitro* Effects of Mutant Strains

No differences in cytotoxicity were observed among the AH-1 wild-type and most of the mutants. Only AH-1::*aopB*, lacking the T3SS, did not induce cytotoxicity in ZF-4 cell culture (data not shown).

## Discussion

One of the most important issues in the study of the virulence factors of pathogenic bacteria is the selection of the experimental model, especially when the obtained results are intended to be extrapolated from the selected model to humans. In the present work, the infection of zebrafish larvae by bath immersion was proposed as an alternative model for the study of the virulence factors of *A. hydrophila*. The great advantage of zebrafish larvae over other models is that experimental infection by bath immersion mimics the natural route of infection. Moreover, injury to the tail also provides a natural portal of entry for the bacteria, mimicking wounds that are frequently used as a portal to spread infection (Janda and Abbott, 2010). Classical experimental intraperitoneal infections in mice and rainbow trout were compared with bath-infected zebrafish larvae using *A. hydrophila* containing several modifications in the virulence factors.

First, the feasibility of the zebrafish larvae infection model to study the virulence factors of *A. hydrophila* was evaluated in experimental infections using the type III secretion system (TTSS) AH-1::*aopB* mutant strain, which was already used in mice and fish (Yu et al., 2004; Sha et al., 2005). The disruption of the T3SS by mutations induced a decrease in bacterial virulence (Vilches et al., 2004, 2008, 2012). The AH-1::*aopB* mutant was less virulent than the wild-type strain when inoculated by bath into zebrafish larvae. This result was in agreement with previous infections in mice and blue gourami, describing lower virulence after intraperitoneal infection with the same mutant strain (Yu et al., 2004; Sha et al., 2005). Interestingly, when the infection was conducted in injured zebrafish larvae, a 48 h delay in mortality was registered. This delay in mortality was not previously observed using vertebrate models (mice and adult fish), but it was described in experimental infections using invertebrate models: the insect *T. molitor* and the crustacean *P. leniusculus* (Noonin et al., 2010). Therefore, when the implications of the T3SS in the virulence of *A. hydrophila* were analyzed, the injured zebrafish larvae model could produce not only the variations previously described in other vertebrate models but also slight differences in mortality kinetics only observed using invertebrate models.

The implications of the surface-associated S-layer in the pathogenesis of *A. hydrophila* were also evaluated in mice and rainbow trout and were compared to zebrafish larvae using the mutant AH-1ΔvapA, which lacks the gene coding for the surface layer protein (Merino et al., 2015). This surface structure is an essential virulence factor of *A. hydrophila*, especially in strains highly virulent to mice and fish (Mittal et al., 1980; Dooley and Trust, 1988; Murray et al., 1988; Noonan and Trust, 1997; Esteve et al., 2004). When the mutant AH-1ΔvapA was intraperitoneally injected into trout and mice and was inoculated into injured zebrafish larvae, offering the bacteria an alternative portal of entry to the body, this mutant was as pathogenic as the wild-type strain. It was described that a double mutant (S-layer and metalloproteases) and a triple mutant (S-layer, metalloproteases, serine protease) were less pathogenic than the wild-type when infecting blue gourami (Yu et al., 2005), suggesting that additional mutations outside of the S-layer were needed to induce a decrease in virulence. Surprisingly when healthy zebrafish larvae were infected, the mutant strain produced less mortality than the wild-type. This result suggested that the S-layer was not totally necessary to the bacteria once it was inside the host, but it contributed to the inflammatory response because it was observed with high expression levels of IL-1β. The implications of the S-layer proteins in the induction of proinflammatory cytokines, such as IL-12p70, TNFα, and IL-1β, have already been described in dendritic and T cells infected with the S-layer mutant *Lactobacillus acidophilus* NCFM (Konstantinov et al., 2008).

The sensitivity of the zebrafish larvae model to minor variations in the pathogenicity of mutant stains when analyzing the function of the different virulence factors was also evidenced in the analysis of the LPS structures. In the present work, two LPS-mutant strains were used: AH-1ΔrmlB, which is devoid of the O-antigen LPS with a complete LPS core (Merino et al., 2015), and AH-1ΔwahD, which lacks the O-antigen LPS and part of the LPS outer core. To our knowledge, this study is the first report describing the pathogenicity of a mutant AH1 strain including residue modifications from the LPS outer core. Regardless of the animal model (mice, trout or zebrafish) and the route of infection (ip injection and bath), the experimental infections with both AH1 mutant strains lacking the O-antigen LPS and the outer core LPS resulted in decreased cumulative mortality. It has been well described that modifications in the O-antigen LPS generated by physical environmental conditions (osmolarity and temperature; Aguilar et al., 1997) or the introduction of specific mutations in *galU*, *galE*, and *gne* of the *A. hydrophila* AH3 O:34 (Canals et al., 2006a, 2007a; Vilches et al., 2007) resulted in a decrease in virulence (Merino et al., 2015). Interestingly, when injured zebrafish larvae were infected, differences in mortality were observed between the mutants AH-1ΔrmlB (lacking of the O-antigen LPS) and AH-1ΔwahD (which lacks the O-antigen LPS and part of the LPS outer-core), which were not previously observed in mice and trout after ip infection. This result suggests that residues from the LPS outer core must be important for virulence. Once again, the injured zebrafish larvae infection model seemed to be more feasible for the study of this virulence factor because ip injection of mice and trout did not enable the detection of slight changes in the bacterial virulence induced by changes in the LPS outer core.

However, the greatest differences depending on the animal model were obtained in the analysis of the polar flagella and the bacterial motility. The *A. hydrophila* strain AH-1 is motile in liquid medium (swimming) through the expression of a polar flagellum, and it produces lateral flagella when grown in semisolid or solid media, being able to swarm. Two mutants were isolated and used: AH-1ΔFlaB-J, a non-polar flagellated mutant unable to swim, and AH-1::motX, which is non-motile but able to produce polar flagella.

A clear effect of motility on the pathogenesis of *A. hydrophila* was observed. AH-1ΔFlaB-J and AH-1::motX were as pathogenic as the wild-type strain when injected into mice and rainbow trout. In these models, the bacteria reached the animal body without using the mutated structures and were able to progress inside the animal, like the wild-type. However, when zebrafish larvae were used, no mortality was registered in either AH-1ΔFlaB-J- or AH-1::motX-infected fish. The bacterial burden of the mutant strains was evaluated to determine the stable presence of bacteria in the fish. Interestingly, the mutant AH-1ΔflaB-J lacking the polar flagellum was not able to replicate inside the fish, while the non-motile mutant strain (AH-1::motX) had the polar flagellum but could not use it. These results suggested that the polar flagellum could be involved not only in motility and adherence but also in bacterial virulence. The lack of flagella or the loss in motility affects the pathogenicity of the bacteria, very likely due to a decrease in adherence to the fish surface to proceed with the infection. This observation was demonstrated using similar *A. hydrophila* mutants (without polar flagella and motility), showing a reduced ability to attach to human epithelial cells and to form biofilms (Gryllos et al., 2001; Canals et al., 2006b, 2007b). Moreover, *A. hydrophila* polar flagellum mutants had less survival and adherence to eel macrophages (Qin et al., 2014).

TLR5 (Toll-like receptor 5) recognizes flagellin by its dominion D1 triggering the activation of proinflammatory and immune genes through the NF-κB (nuclear factor-kappa B) and MAPK (mitogen-activated protein kinase) routes (Hayashi et al., 2001). The AH-1::motX mutant cannot move the flagellum, which can result in low fragmentation of this structure, thus avoiding recognition of the flagellum D0–D1 domains by TLR5. This partial activation of TLR5 could induce low activation of the immune response meditated by this receptor.

The analysis of all of the virulence factors was also conducted in cell culture using ZF-4 cells. No differences in cytotoxicity were observed between mutants and the wild-type strain. Only AH-1::*aopB* lacking the T3SS was unable to induce cytotoxicity, in agreement with previous publications using EPC cells, RAW 264.7 murine macrophages and HT-29 human colonic epithelial cells (Yu et al., 2004; Sha et al., 2005).

This study demonstrates that zebrafish larvae can be used as a simple host model to assess the virulence factors of *A. hydrophila*. This animal model reveals many more differences in pathogenicity than the *in vitro* experiments and enables the detection of slight variations in pathogenesis not previously observed using the classic ip injection of mice or fish.

## Author Contributions

BN, PS, AR, SM, JT, and AF contributed to the conception of the work. PS, AR, and SM acquired and analyzed the data for the work. PS, AR, BN, SM, JT, and AF interpreted the data. PS, AR, and BN drafted the work. AF, AR, JT, SM, and BN revised the article critically for important intellectual content. PS, AR, SM, AF, JT, and BN approved the final version of the article to be published.

## Conflict of Interest Statement

The authors declare that the research was conducted in the absence of any commercial or financial relationships that could be construed as a potential conflict of interest.

## Footnotes

1 http://ec.europa.eu/environment/chemicals/lab_animals/home_en.htm
